# Nutritional assessment and factors associated with malnutrition among the elderly population of Nepal: a cross-sectional study

**DOI:** 10.1186/s13104-019-4282-4

**Published:** 2019-04-30

**Authors:** Man Kumar Tamang, Uday Narayan Yadav, Hassan Hosseinzadeh, Bharat Kafle, Girish Paudel, Saroj Khatiwada, Varalakshmi Chandra Sekaran

**Affiliations:** 1 Department of Nutrition and Dietetics, Central Campus of Technology, Tribhuvan University, Dharan, Nepal; 2 Forum for Health Research and Development, Dharan, Nepal; 3 School of Public Health and Community Medicine, University of New South Wales, Sydney, Australia; 4 Central Department of Population Studies, Tribhuvan University, Kirtipur, Bagmati Nepal; 5 School of Medical Sciences, University of New South Wales, Sydney, Australia; 6 Melaka Manipal Medical College, Manipal Academy of Higher Education, Manipal, India

**Keywords:** Elderly, Malnutrition, Nepal, Physical activity, Polypharmacy

## Abstract

**Objectives:**

This study aimed at assessing the nutritional status among the elderly population and factors associated with malnutrition in the community setting in rural Nepal.

**Results:**

Out of 339 participants, 24.8% (95% CI 20.21–29.30) fell into the normal nutritional status range; 49.6% (95% CI 44.29–54.91) were at risk for malnutrition while 24.8% (95% CI 20.21–29.30) were in the malnourished range, based on Mini Nutritional Assessment scores. Our findings revealed that belonging to a *Dalit* community, being unemployed, having experience of any form of mistreatment, lack of physical exercise, experiencing problems with concentration in past 30 days and taking medication for more than one co-morbidity was significantly associated with the malnutrition status of the elderly.

## Introduction

Every country in the world is facing a demographic challenge due to the drastic growth of population over 60 years. The proportion of this population is projected to grow by 56% between 2015 and 2030, and by 2050 the global proportion of elder populations is projected to be double than its size in 2015. The pace of population aging in many developing countries today is noticeably faster than occurred in developed countries in the past [1, 2].

Nepal, as with other countries in south-east Asia, has been successful in increasing the life expectancy of people. This results in an increased proportion of elder peoples along with social, economic and health problems in the population [3]. The nutritional needs of the elderly population are not recognized as a felt need by the government of Nepal and the working agencies. Nutritional assessment is the systematic process of collecting and interpreting information in order to make decisions about the nature and cause of nutrition related health issues that affect an individual [4].

Adequate diet and nutritional status are important determinants of health among the elderly [5]. Malnutrition in the elderly is defined as a state of faulty or inadequate nutritional status; undernourishment characterized by insufficient dietary intake, poor appetite, muscle wasting and weight loss that causes adverse effects on physiological functions and another clinical outcomes [6] such as decreased quality of life [7, 8], higher infection and complications [9, 10], muscle wasting [8], hospitalization and even higher mortality [8, 10]. There is evidence to support the claim that adequate nutrition can prevent, delay or significantly improve the number of chronic diseases that affect elder people [11, 12]. Research evidence also indicates that nutrition and social support interventions result in better health outcomes among the elderly population and shows that timely interventions can prevent weight changes among those who are at risk of malnutrition [13, 14].

Despite the evidence highlighting the nutrition status of the elderly population in old age home, there continues to be a dearth of evidence examining the nutritional status at the community level in the rural plain region of Nepal. The aim of our study aimed was to assess the nutritional status among the elderly and risk factors associated with malnutrition in the community setting in rural Nepal.

## Main text

### Methods

#### Study designs and participants

This is a community based cross-sectional study among older adults of age 60 and above in the former three Village Development Committee (VDC) of Morang district in eastern Nepal, between August and November 2016. Multi-stage cluster sampling was adopted to select the study subjects with a sample size of 339. Sampling method has been fully described in our previous paper [15].

#### Data collection and study variables

Nutritional assessment was performed with the use of Mini Nutritional Assessment (MNA) tool developed by Nestle Nutrition Institute [16]. Elder mistreatment was measured by the tool developed and published under the authorship of two researchers of this team [15, 17]. The data was collected by trained research assistants having bachelor-level health degree with training in the health sciences after 12 years of schooling. The details on the measurement of independent variables is described in our previous published paper [15].

#### Statistical analysis

The statistical analysis was conducted by the use of the Statistical Package for Social Sciences (SPSS 15.00). The variance inflation factors (VIFs) for all covariates that were included in the logistic regression analysis were less than 2.0. The covariates that were significantly associated (p-value < 0.05) with the dependent variables in univariate analysis were considered in the multivariable analysis for checking the associations.

### Results

#### Study sample characteristics

In total, 339 elderly citizens, 181 males and 158 females participated in the study. The mean age of the male participants was 71.03 (± 8.85) years, ranging from 60 to 110 years. Similarly, the mean age of the female participants was 69.89 (± 8.19) years, ranging from 60 to 115 years. The larger proportion of participants ascribed to Hinduism (96.2%), were married (67.3%), and illiterate (55.8%) and more than 65% of the study subjects revealed that they were employed in some private or government jobs in the past. Among the participants, 12.4% reported current smoking and 17.3% current alcohol use. The majority of the elders (62.2%) lived in joint families and the rest (37.8%) in nuclear families.

#### Nutritional assessment of participants based on MNA tool

Of the total, 25.6% (95% CI 21.06–30.34) elderly fell into normal range of nutritional status; 49.6% (95% CI 44.29–54.91) were at risk of malnutrition and, 24.8% (95% CI 20.21–29.30) were in the malnourished range in accordance with the MNA scores (Table 1).

**Table 1:** Prevalence of malnutrition among the older population of eastern Nepal

Nutritional status	Prevalence in females n (%)	Prevalence in males n (%)	Overall prevalence n (%)	95% CI of overall prevalence
Malnutrition	50 (31.6)	34 (18.8)	84 (24.8)	20.21–29.30
At risk of malnutrition	72 (45.6)	96 (53.0)	168 (49.6)	44.29–54.91
Malnourished	36 (22.8)	51 (28.2)	87 (25.6)	21.06–30.34

#### Factors associated with malnutrition status

Sex, gender, educational status, ethnicity, occupation, the income level of caregiver, smoking status, smoking status, the experience of any mistreatment, daily physical activity, having concentration problems and poly-pharmacy were the studied independent variables. The covariates had p-value < 0.05 in univariate analysis were considered in the multivariable analysis.

Independent factors associated with malnutrition status among the elderly population after controlling for confounders on bivariate analysis, performing a backward elimination procedure. The results are presented in Table 2. The likelihood of being malnourished was 2.69 [OR = 2.69, 95% CI 1.17–6.21] times higher among the Dalits community (backward untouchable caste according to traditional Hindu caste system) than those who belonged to a higher caste in Nepal. The unemployment status among the elderly had a significant impact on the malnutrition status of the elderly population. It was found that those who unemployed were 3.23 times [OR = 3.23, 95% CI 1.63–6.41] more likely to be malnourished. The analysis showed that for elders, the chances of falling into the malnutrition range increased by 4.05 times (OR = 4.05, 95% CI 1.90–8.60) if they experienced any forms of mistreatment from caregivers/family members.

**Table 2:** Factors associated with malnutrition among the elder population of rural eastern Nepal

Variables	Unadjusted OR (95% C.I)	Adjusted OR (95% C.I)
Ethnicity		
Upper caste (Chhetri/Brahmin)	1	1
Dalit (backward caste in traditional Hindu caste system)	2.30 (1.22–4.44)	2.69 (1.17–6.21)
Madhesi (people of plain land origin)/indigenous caste (Janjati)	0.88 (0.49–1.57)	1.02 (0.50–2.08)
Occupation		
Employed	1	1
Unemployed	2.94 (1.76–4.90)	3.23 (1.63–6.41)
Experience of any mistreatment		
No	1	1
Yes	5.80 (2.94–11.46)	4.05 (1.90–8.60)
Daily physical activity		
Yes	1	1
No	6.00 (2.65–13.55)	4.67 (1.87–11.66)
Having concentration problems		
No	1	1
Yes	3.06 (1.80–5.19)	2.71 (1.45–5.07)
Polypharmacy (Taking regular medication for more than one disease)		
No	1	1
Yes	3.17 (1.74–5.77)	3.01 (1.53–5.92)

Elders who were not involved in daily physical activity [OR = 4.67, 95% CI 1.87–11.66], having concentration problems in the past 30 days [OR = 3.01, 95% CI 1.53–5.92] and those taking medication for more than one co-morbidity, i.e., polypharmacy [OR = 3.01, 95% CI 1.53–5.92] had higher risk of falling into the malnutrition range.

### Discussion

Nutritional status of older people results from a complex interplay between dietary, socio-economic, physical and psychological factors [18]. Long recognized as public health problem hidden from the public eye, now there is revived interest and considerably more scientific attention in recent years, as evidenced by a number of scientific publications [19]. Addition of extra years is marked by declining health, reduced mobility, isolation, loneliness, change is self-care behaviors that sometimes lead to functional disability and need of support [20, 21].

Our study has revealed that 24.8% of rural elderly people are malnourished and 49.6% of elderly are at risk of malnutrition. Thus, it appears that malnutrition is much higher among the Nepalese elderly residing in the rural community. This finding is consistent with the results of earlier studies conducted in Nepal, India and Bangladesh [20–22]. Consistent with our findings, the study from Nepal and India [20, 21] showed prevalence of malnutrition to be higher among females compared to their male counterparts; however, these findings were not significant. In this light, Nepal ranks 115th position in gender inequality index in the world [23], which clearly presents the picture of gender inequality existing in the country which possibly reflects the disparity of health-related outcomes across the genders. Observing the high heterogeneity of malnutrition prevalence, a gender-wise approach could be the good step to address this problem.

Our findings showed that elderly from the Dalit community were at more than two times at higher risk of malnutrition compared to elders of a higher caste. Our study aligns with findings from study conducted in Nepal [20] where ethnicity was identified as a risk factor for malnutrition at the community-level. Most members of Dalit ethnic group belong to the lower socio-economic status, have limited access to health, education, employment, and other resources. A total of 42% of the elders from Dalit community fall below the poverty line which is 17% higher than that 25.2% of the national average [24]. They have long been neglected and ignored in the social milieu and suffered cumulative domination. Additionally, the Nepal Multidimensional Social Inclusion Index shows that 46% of the Terai origin Dalit do not consume adequately nutritious food throughout the year [25].

In the current study, unemployed elderly population were at 3.23 times more risk of malnutrition compared to those who were employed. This can be explained by the fact that the employed elderly possibly could have good financial status, resulting in good access to nutritional foods. In addition, they have to depend economically less on the caregivers for the fulfillment of dietary and basic needs needed for good health. Consistent with our findings, study from Egypt [26] have identified unemployment as a risk factor for malnutrition among the elders. Regarding the experience of any mistreatment, we found that elders who experienced some forms of mistreatment in the last 3 months had odds of 4.03 times more risk of malnutrition compared to those who never experiences mistreatment. Interestingly, this is probably the first study from our setting that established the temporal association between elder mistreatment and malnutrition. Elder mistreatment has an array of negative sequelae beyond traumatic injury and pain [27]. In Nepalese society, elder abuse is associated with a degree of dependency, the temperament of the caregivers, low income [17] and studies from other settings [28, 29] have evidenced this phenomenon. This can be explained by the fact, abused elders may lose sleep, stop or skip eating and develop stress disorders, which in turn may bring negative outcome like weakness, nutrition and hydration issues, thus deteriorating the health of the elderly. Taken together, caregiver counseling, privileged social security and nutrition support program could benefit the elders.

In this study, the elderly who reported a lack of physical activity were at 4.67 times likelihood of being malnourished, as has been reported in other studies [30, 31]. This can be explained by the fact that lack of physical activity can lead to lifestyle disease including both physical and mental health problems. It is well documented that physical inactivity, or a decreased physical activity level is an underlying mechanism of sarcopenia [32] and therefore, promotion of physical activity with nutrition intervention could be important for improving the well-being of elders. In our study sample, the elderly who experienced concentration problems within the past 30 days were 3.01 times more likely to be malnourished compared to their counterparts. This logic behind this could be that elderly who could not recall or forgot the location of the placed food items, money or other belongings etc., might not have been able to fulfill the daily nutrition needs. In Nepalese society, most of the times elders have some hard cash to fulfill their own needs but in case if they forget, they don’t share their concerns with caregivers. In this light, literature has also clearly identified a decline in cognitive capacity as a risk factor for elder malnutrition [11].

The elderly who had history of polypharmacy were at 3.01 times more risk of malnutrition compared to their counterparts who did not had history of polypharmacy. The could be explained by the fact that, polypharmacy is chosen by the practitioners for the treatment of such multiple co-morbidities, can indirectly result in malnutrition because of side effects like poor appetite, nausea, dry mouth and constipation. Evidence shows that moderate intakes have a protective effect on malnutrition while an excessive intake may have a negative effect on the health of elders [33]. This finding suggests the need for longitudinal exploration of the status of self-prescribed medicines, compliance with standard drug regime and, its effect on malnutrition status of elderly. The interplay and coordination between a multidisciplinary team with promising nutritional counseling could improve their nutritional status, in turn; adverse outcomes like hospitalizations, complications, and mortality can be reduced [34].

## Limitations

A limitation of this study is the cross-sectional design, no conclusions can be drawn in line with the cause-effect model. In addition, the study was conducted in one district, so its findings could not be generalized to other setting of Nepal.

## Abbreviations

MNA: Mini Nutritional Assessment
VDC: Village Development Committee
SPSS: Statistical Package for Social Sciences (SPSS
CI: confidence interval
VIFs: variance inflation factors
OR: odds ratio
